# Effects of Small Molecule Calcium-Activated Chloride Channel Inhibitors on Structure and Function of Accessory Cholera Enterotoxin (Ace) of *Vibrio cholerae*

**DOI:** 10.1371/journal.pone.0141283

**Published:** 2015-11-05

**Authors:** Tanaya Chatterjee, Irshad Ali Sheikh, Devlina Chakravarty, Pinak Chakrabarti, Paramita Sarkar, Tultul Saha, Manoj K. Chakrabarti, Kazi Mirajul Hoque

**Affiliations:** 1 Department of Biochemistry, Bose Institute, P1/12 CIT Scheme VIIM, Kolkata, 700054, India; 2 Division of Molecular Pathophysiology, National Institute of Cholera & Enteric Diseases, P-33, CIT Road, Scheme XM, Beliaghata, Kolkata, 700010, India; Università degli Studi di Milano, ITALY

## Abstract

Cholera pathogenesis occurs due to synergistic pro-secretory effects of several toxins, such as cholera toxin (CTX) and Accessory cholera enterotoxin (Ace) secreted by *Vibrio cholerae* strains. Ace activates chloride channels stimulating chloride/bicarbonate transport that augments fluid secretion resulting in diarrhea. These channels have been targeted for drug development. However, lesser attention has been paid to the interaction of chloride channel modulators with bacterial toxins. Here we report the modulation of the structure/function of recombinant Ace by small molecule calcium-activated chloride channel (CaCC) inhibitors, namely CaCC_inh_-A01, digallic acid (DGA) and tannic acid. Biophysical studies indicate that the unfolding (induced by urea) free energy increases upon binding CaCC_inh_-A01 and DGA, compared to native Ace, whereas binding of tannic acid destabilizes the protein. Far-UV CD experiments revealed that the α-helical content of Ace-CaCC_inh_-A01 and Ace-DGA complexes increased relative to Ace. In contrast, binding to tannic acid had the opposite effect, indicating the loss of protein secondary structure. The modulation of Ace structure induced by CaCC inhibitors was also analyzed using docking and molecular dynamics (MD) simulation. Functional studies, performed using mouse ileal loops and Ussing chamber experiments, corroborate biophysical data, all pointing to the fact that tannic acid destabilizes Ace, inhibiting its function, whereas DGA stabilizes the toxin with enhanced fluid accumulation in mouse ileal loop. The efficacy of tannic acid in mouse model suggests that the targeted modulation of Ace structure may be of therapeutic benefit for gastrointestinal disorders.

## Introduction

The diarrheal disease cholera caused by Gram negative bacteria *Vibrio cholerae* is still a potential threat in many developing countries [1]. Pathogenesis of cholera occurs due to synergistic effect of a number of toxins produced by *V*. *cholerae* [2, 3]. Amongst various toxins released by *V*. *Cholerae*, Ace is a classic enterotoxin, which comprises the “virulence cassette” along with cholera toxin (CTX) and zonula occludens toxin (Zot). Ace, a small integral membrane protein (M.W. 11.3 kDa, Swiss-Prot entry C3M620-1), contributes to fluid accumulation in ligated rabbit ileal loops and increases short circuit current (*I*_*sc*_) in Ussing chambers in a concentration dependent manner [4, 5]. Ace is known to activate calcium-dependent chloride-bicarbonate secretion in human colonic carcinoma T84 cell line [6]. We have previously reported that Ace potentiated ATP stimulated chloride secretion in T84 cells *via* stimulation of calcium-activated chloride channel (CaCC) [7]. Ubiquitous expression of CaCCs in both epithelial and non-epithelial cells, along with their involvement in broad range of biological functions, especially fluid secretion in intestinal cells, make CaCCs potential drug targets for secretory diarrhea [8].

Administration of oral rehydration solution (ORS) still remains the first-line therapy for the treatment of secretory diarrhea. However, as much as this basic approach is substantially effective in most cases of diarrhea, ORS does not reduce frequency, stool volume or the duration of the disease. In this context, adjuvant therapy to rehydration, such as micronutrient supplementation (zinc), probiotics, or antisecretory agents may offer a safe complement to ORS to reduce the severity of the symptoms. The identification of small molecule inhibitors targeting chloride channels may serve as an alternative approach to combat diarrhea. High-throughput screening has revealed different chemical classes of small-molecule inhibitors of chloride channels, some with low nanomolar affinity and chloride channel selectivity, and of higher potency than the inhibitors reported earlier [9,10]. These small-molecule inhibitors should serve as drug development candidates to study the role of chloride channels in diarrhea and other gastrointestinal disorders, and also in tissues where these channels are expressed. However, the major challenge still remains in the clinical development of chloride channel inhibitors with appropriate pharmacological properties, which upon interaction with bacterial toxin can perturb its structure. Along this line, our present paper aims to investigate the effect of small molecule chloride channel inhibitors such as CaCC_inh_-A01 (PubChem CID: 2898877), digallic acid (PubChem CID: 341) and tannic acid (PubChem CID: 16129778) (S1 Fig) on the structure of Ace and the resultant modulation of its function. The CaCC inhibitors chosen in the present study have been previously identified by high throughput screening [11]. Literature reports inhibitory effect of CaCC_inh_-A01 using human intestinal HT-29 cells [12]. CaCC_inh_-A01 is also known to inhibit ANO1 (synonymously, TMEM16A), a calcium-activated chloride channel, which is expressed and amplified in human cancers [13]. The other two small molecules, DGA and tannic acid have also been identified as CaCC inhibitors [14]. In the USA, Cesinex^®^, a tannic acid based medical food, comprising mainly of food grade tannic acid from plant source along with dried chicken egg white albumin, is used for the treatment of diarrhea [15].

A recent patent reported administration of Ace to patients suffering from cystic fibrosis (CF), a genetic disorder, by increasing chloride secretion within the lungs [16]. Because of the identification of Ace as a potent candidate both for CF and secretory diarrhea, our objective was to study the interaction of Ace with CaCC inhibitors and the structural alteration caused thereof using various biophysical methods. Small molecule induced structural alteration of Ace was explored using tryptophan fluorescence and far-UV CD. The binding constants for the Ace-CaCC complexes, along with the standard free energy for the association, have been estimated using quenching of tryptophan fluorescence. Functional studies of Ace induced by CaCC inhibitors were performed by Ussing chamber and mouse ileal loop experiments.

Proteins are dynamic, and the large numbers of internal motions result in conformational changes affecting their function [17]. Here, experimental methods probing the interactions between Ace and the CaCC inhibitors have also been validated by computational methods, employing docking followed by molecular dynamics (MD) simulation. CaCC_inh-_A01 and DGA were docked to Ace to understand the similarities and differences between their binding modes. MD simulations of Ace-CaCC inhibitor complexes were then performed highlighting the interaction between the protein surface and the inhibitors. The structural alteration in terms of root mean square deviations has been found to be greatest for the unbound Ace, followed by the complexes formed by DGA and CaCC_inh_-A01.

To the best of our knowledge the targeted approach to modulate the structure of Ace by small molecules CaCC inhibitors has not been reported so far. Considering the contribution of Ace in the pathogenesis of cholera, before the onset of slow acting CTX, and its targeted role in the treatment of diarrhea and CF, these results may have implications for the development of chloride channel based drug therapy.

## Materials and Methods

### Ethics statement

All animal work and care were performed according to the guidelines established by the National Institute of Cholera and Enteric Diseases (NICED) ‘Institutional Animal Ethics Committee’ (IAEC) concerned under the guidance of the ‘Committee for the Purpose of Control and Supervision of Experiments on Animals’ (CPCSEA), Govt. of India. Animal experiments were approved by NICED ‘IAEC’ (approval no. Apro/75/24/11/2010). All reasonable efforts were made to ameliorate suffering, including anesthesia for painful procedures.

### Materials

*V*. *cholerae* classical strain O395 was a gift from Dr. R. Nandi, NICED. Urea, IPTG and PMSF were purchased from Sigma Chemicals (Mumbai, India). Ni-NTA Superflow Agarose was procured from Qiagen. CaCC_inh_-A01, DGA and tannic acid were purchased from Calbiochem, Santa Cruz Biotechnology, Inc. and Sigma, USA, respectively. All other chemicals obtained from Merck (Mumbai, India) were of analytical grade.

### Purification of recombinant Ace

Recombinant Ace protein was expressed into *E*. *coli* M15 (pREP4) cells and purified using Ni-NTA affinity chromatography as described previously [7]. The purified recombinant protein was run into 10% tris-tricine gel followed by silver staining. Protein concentration was estimated by spectrophotometric method using the extinction coefficient of 26470 M^-1^cm^-1^ (http://expasy.org/sprot/) at 280 nm.

### Fluorescence spectroscopy

Steady state fluorescence was measured in a Hitachi F-3010 spectrofluorimeter in quartz cuvette of 1 cm path length and a scanning speed of 240 nm per min. The band passes for both excitation and emission was kept at 5 nm. For tryptophan fluorescence, the sample was excited at 295 nm and emission was recorded in the range of 310 to 420 nm. For fluorescence experiment, protein concentration of 5 μM was used in 0.1 M potassium phosphate buffer (pH 8.0).

In order to compare the stability for native Ace with those of Ace-small molecule complexes (Ace-small molecule molar ratio = 1:1), urea-induced denaturation was carried out. A 10 M freshly prepared solution of urea was made and samples were incubated at 25°C for 3 h with different concentration of urea solutions.

### Circular dichroism spectroscopy

Circular dichroism spectroscopy was recorded in a JASCO J800 spectropolarimeter using 1 mm path length cuvette. Far-UV CD experiments were carried out using protein concentration of 5 μM in 0.1 M potassium phosphate buffer (pH 8.0) in the wavelength range of 200–260 nm, with a step resolution of 1 nm. The content of DMSO (used as solvent) never exceeded 1.5% (v/v) during CD measurements. CDNN software tool was used for the deconvolution of far-UV CD spectra [18]. CD data for each sample were averaged from three different observations, each of which was obtained from five repeat scans. Deconvolution of CD spectra was carried out using the experimental spectral range for the protein with 106 residues and 12.6 kDa molecular weight.

Temperature induced denaturation of protein results from the weakening of hydrogen bonding and other stabilizing interactions [19]. For the thermal unfolding of Ace in presence and absence of small molecules, far-UV CD spectra were recorded as a function of temperature between 20 and 70°C in steps of 2°C with an equilibration time of 2 min at each temperature. The observed ellipticities were converted into the mean residue ellipticities [*θ* deg.cm^2^.dmol^-1^] which is given by

$$[\theta_{222}]=100\theta Mw/cln$$ (1)

where *θ*_*222*_ is the measured ellipticity in degrees, *c* is the protein concentration in mg/mL, *l* is the path length in cm, *M*_w_ is the molecular weight of Ace and *n* is the number of amino acid residues of Ace. The Gibbs free energy of unfolding is given by

$$\Delta G=-RT\text{ln}\left(\frac{\theta -\theta_{U}}{\theta_{F}-\theta }\right)$$ (2)

where *θ* is the observed ellipticity at any temperature *T*, *θ*_*F*_ is the ellipticity of the fully folded form and *θ*_*U*_ is the ellipticity of the unfolded form. The temperature dependence of the secondary structure of Ace after binding to CaCC inhibitors was estimated by plotting [*θ*_*222*_] as a function of temperature *T*, using Gibbs-Helmholtz equation

$$\Delta G=\Delta H\left(\text{1}-T/T_{M}\right)-\Delta C_{p}T_{M}\left[\text{1}-\left(T/T_{M}\right)+\left(T/T_{M}\right)\text{ln}\left(T/T_{M}\right)\right]$$ (3)

where *T*_*M*_ is the melting temperature, Δ*H* is the change in enthalpy and Δ*C*_*p*_ is the change in specific heat capacity from the folded to the unfolded state.

### Binding of small molecule CaCC inhibitors with Ace

Stock solutions of CaCC_inh_-A01 and DGA were prepared by dissolving the solid samples in DMSO, whereas the stock solution of tannic acid was prepared by dissolving it into Mili Q water. Quenching of tryptophan fluorescence of Ace was used to measure the binding affinity of small molecule CaCC inhibitors to the protein. The fluorescence quenching was monitored after exciting the protein at 295 nm and measuring the emission at 340 nm. All experiments were carried out in triplicates. A plot of *F*_0_/*F*_c_ versus varying concentration of CaCC inhibitors was plotted and analyzed according to the Stern-Volmer equation given below

$$\frac{F_{\text{0}}}{F_{C}}=\text{1}+K_{SV}[Q]$$ (4)

where *F*_*0*_ is the fluorescence intensity of the protein without small molecules, *Fc* is the corrected fluorescence intensity in the presence of a given quencher concentration [*Q*], and *K*_SV_ is the Stern-Volmer constant. The slopes of the linear plots give the values of *K*_SV_. The binding constants for Ace complexed with small molecule inhibitors were determined, by using the following equation

$$\text{log}\left[\frac{F_{0}-F_{c}}{F_{c}}\right]=\text{log}(K)+n\text{log}[Q]$$ (5)

where *F*_*0*_, *F*_*c*_ and [*Q*] are the same as the parameters in the Stern-Volmer equation, *K* is the binding constant and *n* is the substantive binding sites on the surface of the protein to accommodate the ligand molecules.

### Animal experiments

The ileal loop experiment was performed by a modified rabbit ileal loop assay originally described by De and Chatterje [20]. The C57BL/6 male mice, 6–8 weeks of age were used for ileal loop experiments [21]. Briefly, mice starved for 24 h with free access to water and anesthetized with intraperitoneal administration of a mixture of ketamine (35 mg/kg of body weight) and xylazine (5 mg/kg of body weight). A laparotomy was performed, and the experimental loops of 4-cm length were constricted at the terminal ileum by tying with non-absorbable silk thread. Loops were instilled with 120 μl PBS containing Ace in the presence or absence of chloride channel inhibitors in a 1:1 molar ratio by means of a tuberculin syringe fitted with a disposable needle through the ligated end of the loop as the ligatured was tightened. The intestine was returned to the peritoneum; the mice were sutured and allowed to recover from anesthesia. Thereafter they were returned to their cages without any analgesics treatment and monitored every hour for any unexpected consequence due to surgery. Death rarely occurred during or after recovery from anesthesia. All mice that survived beyond 6 h time point were only considered for analysis. After 6 h, these animals were sacrificed by cervical dislocation, and the loops were excised. The fluid from each loop was collected, and the ratio of the amount of fluid contained in the loop with respect to the length of the loop (fluid accumulation ratio in g/cm) was calculated as a reflection of the efficacy of Ace. Prior approval for this study and animals were handled in accordance with the guidelines of the Institutional Animal Ethics Committee.

### Measurement of short-circuit current (*I*_*SC*_) using Ussing chamber

Adult C57BL/6 male mice of body weight 25–35 were used for Ussing chamber experiments. The distal ileum removed from anesthetized mice was placed in HCO_3_^−^-free Ringer solution that contained (in mM) 140 NaCl, 5 KCl, 1 MgCl_2_, 2 CaCl_2_, 10 HEPES, and 4 glutamine, pH 7.4. Mice were euthanized following the removal of the distal ileum. The ileum was partially stripped of serosal and muscle layers, and the mucosa was mounted in lucite chambers and bathed at 37°C on both sides with HCO_3_^−^-free Ringer solution, which was continually circulated with a gas lift oxygenated with 100% O_2_. The mouse ileum was allowed to equilibrate for at least 30 min prior to the experiments, following which various CaCC inhibitors [CaCC_inh_-AO1 (5 μM and 100 μM) or tannic acid (5 μM and 100 μM)] were added to the luminal solution and incubated for 30 min before being stimulated with 5 μM Ace. Transepithelial potential differences were clamped to 0 mV using a VCC MC6 multichannel voltage current clamp amplifier (Physiologic Instruments) as described previously [21]. The short-circuit current (*Isc*) needed to clamp the transepithelial potential at 0 mV was recorded using Ag-AgCl electrodes in 3 M KCl agar bridges. The change in *Isc* induced by luminal Ace treatment was expressed as the difference in *Isc* before and after Ace addition. The viability of epithelia was examined by the mucosal addition of 10 mM glucose at the end of each experiment.

### Statistical analyses

Results are expressed as means ± SE of at least six independent experiments. Paired and unpaired t tests were used by origin software (OriginLab, MA) to compare mean values within one experimental series. In the fluid accumulation assay and short-circuit current measurement, ANOVA with post hoc Bonferroni’s test was used to assess statistical significance. P<0.05 was considered to be statistically significant.

### Docking and simulation

As earlier reported, Ace protein had been modelled using a template structure, zhaoermiatoxin from *Zhaoermia mangshanensis* [Protein Data Bank (PDB) entry 2PH4], a dimeric toxin which showed reasonable sequence similarity (45%) with the target [7]. The protein is dimeric with two helices on each subunit (H1, H2 and H1’, H2’); the structure, its validation, along with the sequence alignment with template are provided in S2 Fig. Docking was performed using the modelled structure and the two inhibitors, CaCC_inh_-A01 and DGA. The third molecule, tannic acid was excluded because of its bulky size compared to the binding site of the protein. Autodock 4 [22] was employed to generate the docked structures; the ones containing optimum number of hydrogen bonds (checked by HBPLUS [23]) and interactions (checked by favourable binding energy obtained from Autodock) were selected. Moreover, to justify the experimental quenching of tryptophan fluorescence, the proximity of the ligand to Trp residues was also a consideration. The complex identified was further subjected to MD for 20 ns after minimisation and equilibration. The sander module of AMBER 10.0 software package [24] was used for all simulations in explicit water at 300 K with the FF99SB force field parameters. Solvation of protein was carried out using TIP3 Box. For minimisation of the molecules, steepest descent for 500 cycles was used, after which conjugate gradient method was employed for 20,000 cycles before the production run. The system heating was carried out 300 K at constant volume conditions (NVT) within 40 ps and equilibrated following minimisation. The SHAKE algorithm was used to constrain the bonds involving hydrogen. Constant pressure (NPT) periodic boundary conditions were employed for the production run of 20 ns duration. For Lennard-Jones interactions cut off distance was set to 12 Å, along with 2 fs of integration time step. Particle Mesh Ewald method was employed to calculate the electrostatics interactions. The coordinates were saved after each 2 ps. The ptraj module of AMBER was used for the analysis of the trajectories. For the parameterization of the ligand, PRODRG server [25] [http://davapc1.bioch.dundee.ac.uk/cgi-bin/prodrg/] was used initially to add hydrogen to the structures downloaded from PubChem [https://pubchem.ncbi.nlm.nih.gov/]. Then antechamber from Ambertools was employed for atom type assignment and charge generation, the parameters were loaded through the help of AMBER force field (GAFF) for rational drug design [26,27].

To gain insight into the protein-ligand binding energetics, the docked complexes after 20 ns of simulation in explicit water were used to calculate the binding energy. For computation of binding energies of the small molecules, the molecular mechanics generalized Born surface area (MMGBSA) approach [28] was employed by taking the last 5 ns data from the trajectory. The scripts in the AMBER 10 package were used to calculate the interaction energies. The difference between the free energies of the receptor-ligand complex (*ΔG*_*complex*,*solvated*_) and the unbound receptor (*ΔG*_*receptor*,*solvated*_) and ligand (*ΔG*_*ligand*,*solvated*_) provides the free energy of binding of the receptor to the ligand and can be given as following

$$\Delta G_{binding,solvated}=\Delta G_{complex,solvated}-\;[\Delta G_{receptor,solvated}+\Delta G_{ligand,solvated}]$$

Here, *G*_*solvated*_ for each of the three states consists of two terms, *G*_*polar*_ (estimated by Generalized Born equation) and *G*_*nonpolar*_ (an empirical term for the hydrophobic contribution, calculated from solvent accessible surface area), omitting the contribution due to entropy.

## Results

### Urea induced unfolding studies of Ace and its complexes with small molecules

**Fig 1:**
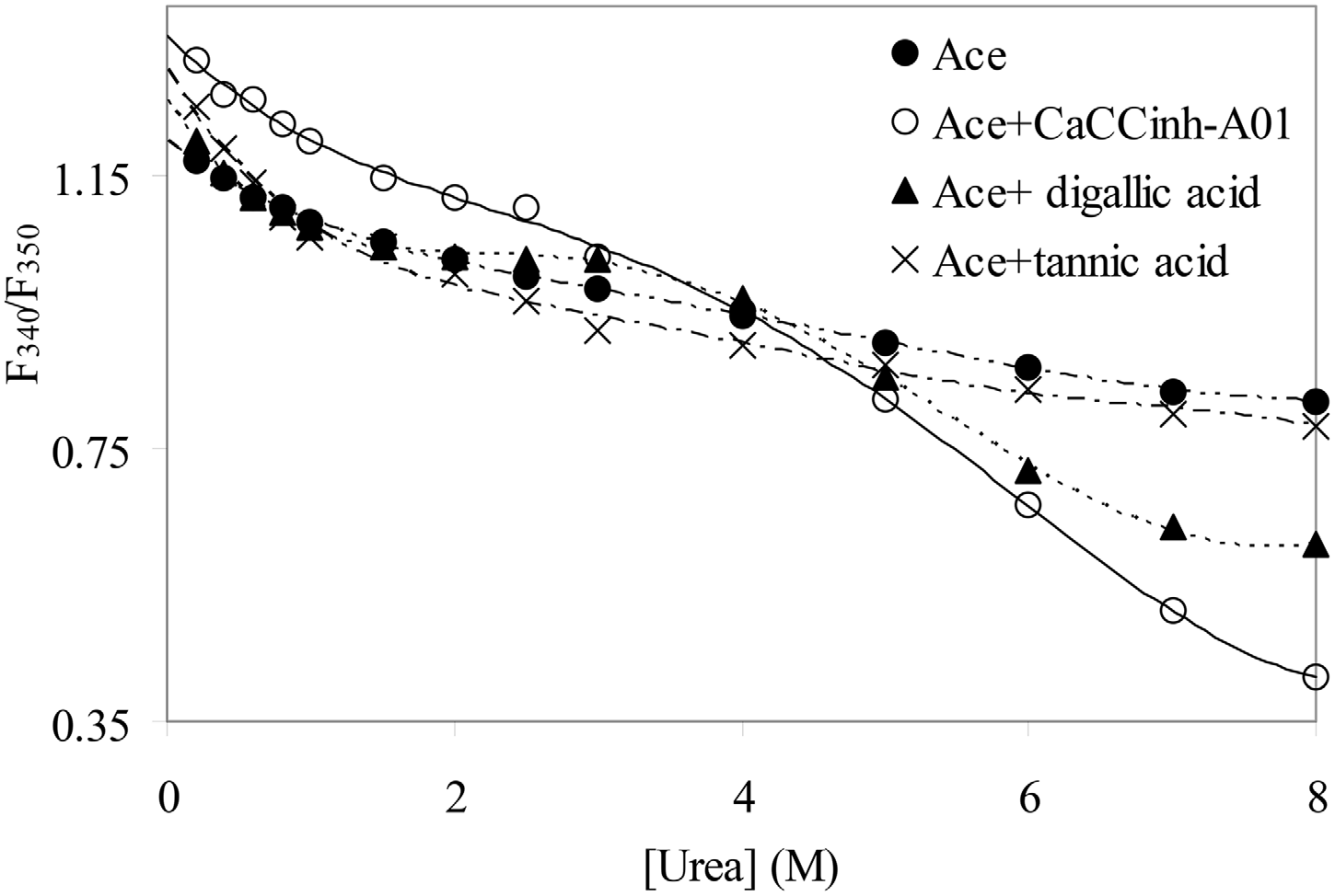
Urea induced denaturation of Ace and Ace complexed with CaCC inhibitors (1:1 molar ratio) at 25°C. The protein concentration used was 5 μM in 0.1 M potassium phosphate buffer pH 8.0. The unfolding was measured from the ratio of fluorescence intensities (F_340_/F_350_) after exciting the protein at 295 nm at different urea concentrations.

The urea induced unfolding studies of Ace and its complexes with CaCC inhibitors were monitored by tryptophan fluorescence by exciting the protein at 295 nm. The ratio of fluorescence intensities F_340_/F_350_ for native Ace and Ace bound to CaCC inhibitors were plotted against varying concentrations of urea and data were fitted using a two state *F*→*U* model, where *F* is the folded and *U* is the unfolded state, respectively (Fig 1).

To analyze the unfolding data the following equation was used

$$S=\frac{S_{F}e^{\left(\frac{\Delta G_{FU}}{RT}\right)}+S_{U}}{e^{\left(\frac{\Delta G_{FU}}{RT}\right)}+1}$$ (6)

where *S* is the ratio of fluorescence intensities (F_340_/F_350_) at each urea concentration. The unfolding free energy (*ΔG*_*FU*_ *)* at each urea concentration was analyzed using the equation:

$$\Delta G_{FU}=\Delta G_{FU}^{H_{2}O}-m_{FU}{\left[dFU\right]}_{1/2}$$ (7)

where *m*_FU_ is the dependency of the *ΔG*_*FU*_ on urea concentration, and *ΔG*_*FU*_^*H*^_*2*_^*O*^ is the change in free energy in the absence of urea. The midpoint of transition, [*d*_FU_]_1/2_ was obtained by dividing *ΔG*_FU_ by the slope.

The unfolding free energy *ΔG*_FU_ increases upon binding with CaCC_inh_-A01 and DGA, compared to native Ace, whereas binding of tannic acid leads to instability of the protein (Table 1). The mid point of transition [*d*_*FU*_]_1/2_ was found to be 3.1 M for native Ace which increased to 3.8 M and 3.2 M for CaCC_inh_-A01 and DGA, respectively. For tannic acid [*d*_*FU*_]_1/2_ was found to be 1.95 M, much less as compared to native Ace. The fluorescence emission spectra of Ace-tannic acid complex showed red shift compared to native Ace, indicating exposure of tryptophan residue towards more polar solvent (data not shown).

**Table 1:** Urea induced unfolding of Ace and Ace complexed with various CaCC inhibitors^†^.

	*ΔG*_FU_ (kcal.mol^-1^)	*m*_FU_ (kcal.mol^-1^.M^-1^)	[*d*_FU_]_1/2_ (M)
**Ace**	1.06 ± 0.2	0.34 ± 0.076	3.14 ± 1.04
**Ace + CaCC**_**inh**_ **-A01**	1.45 ± 0.26	0.38 ± 0.07	3.80 ± 1.01
**Ace + DGA**	1.29 ± 0.3	0.40 ± 0.08	3.16 ± 0.86
**Ace + tannic acid**	0.59 ± 0.26	0.30 ± 0.07	1.95 ± 0.96

^**†**^Based on data shown in Fig 1.

### Conformational changes of Ace induced by small molecule CaCC inhibitors

**Fig 2:**
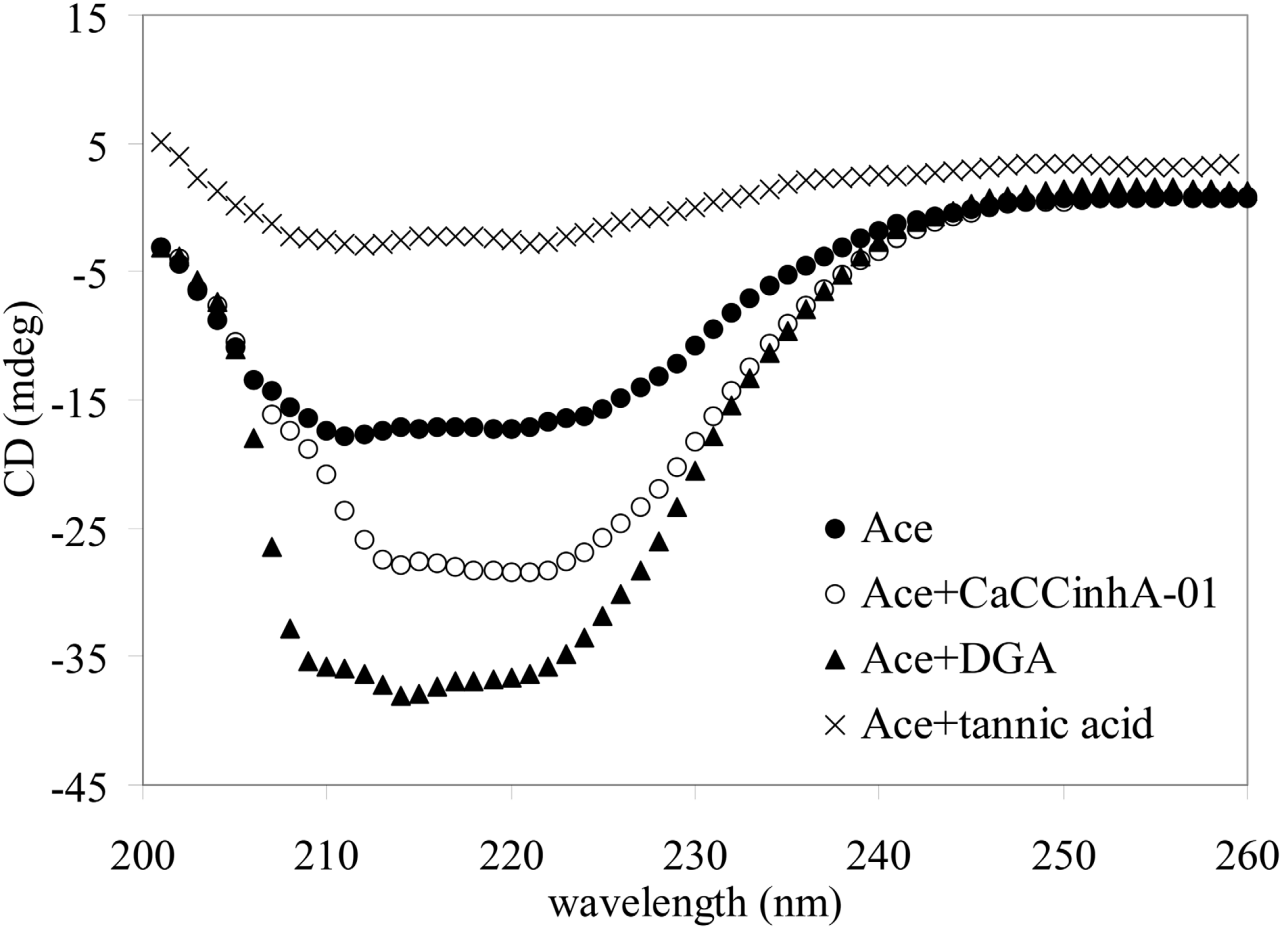
Far-UV CD of Ace and Ace complexed with CaCC inhibitors. Spectra were measured in 0.1 M potassium phosphate buffer (pH 8.0) with protein-ligand molar ratio of 1:1 at 25°C.

To probe the conformational changes of Ace after binding to small molecules, far-UV CD was carried out. The secondary structural contents of Ace in the absence and presence of different CaCC inhibitors are given in Table 2. The α-helical content of Ace-CaCC_inh_-A01 and Ace-DGA complexes increased compared to native Ace with the concomitant reduction of random coil (Fig 2). However, binding to tannic acid leads to reduction of α-helical content compared to native Ace, indicating the loss of protein secondary structure.

**Table 2:** Secondary structural content of native Ace and Ace-CaCC inhibitor complexes^†^.

	Ace alone	Ace + tannic acid	Ace + DGA	Ace + CaCC_inh_-A01
**θ**_**222**_	-17.3 ± 0.8	-3.5 ± 0.8	-26.5 ± 0.7	-36.0 ± 0.5
**α-helix (%)**	43.0 ± 2.7	21.3 ± 1.6	55.4 ± 1.1	63.7 ± 1.8
**Random coil (%)**	26.5 ± 2.6	44.2 ± 1.5	19.7 ± 0.8	15.8 ± 0.7

^†^Data were deconvoluted using CDNN software (http://thelab.photophysics.com/circular-dichroism/protein-secondary-structure-analysitools-cdnn/).

**Fig 3:**
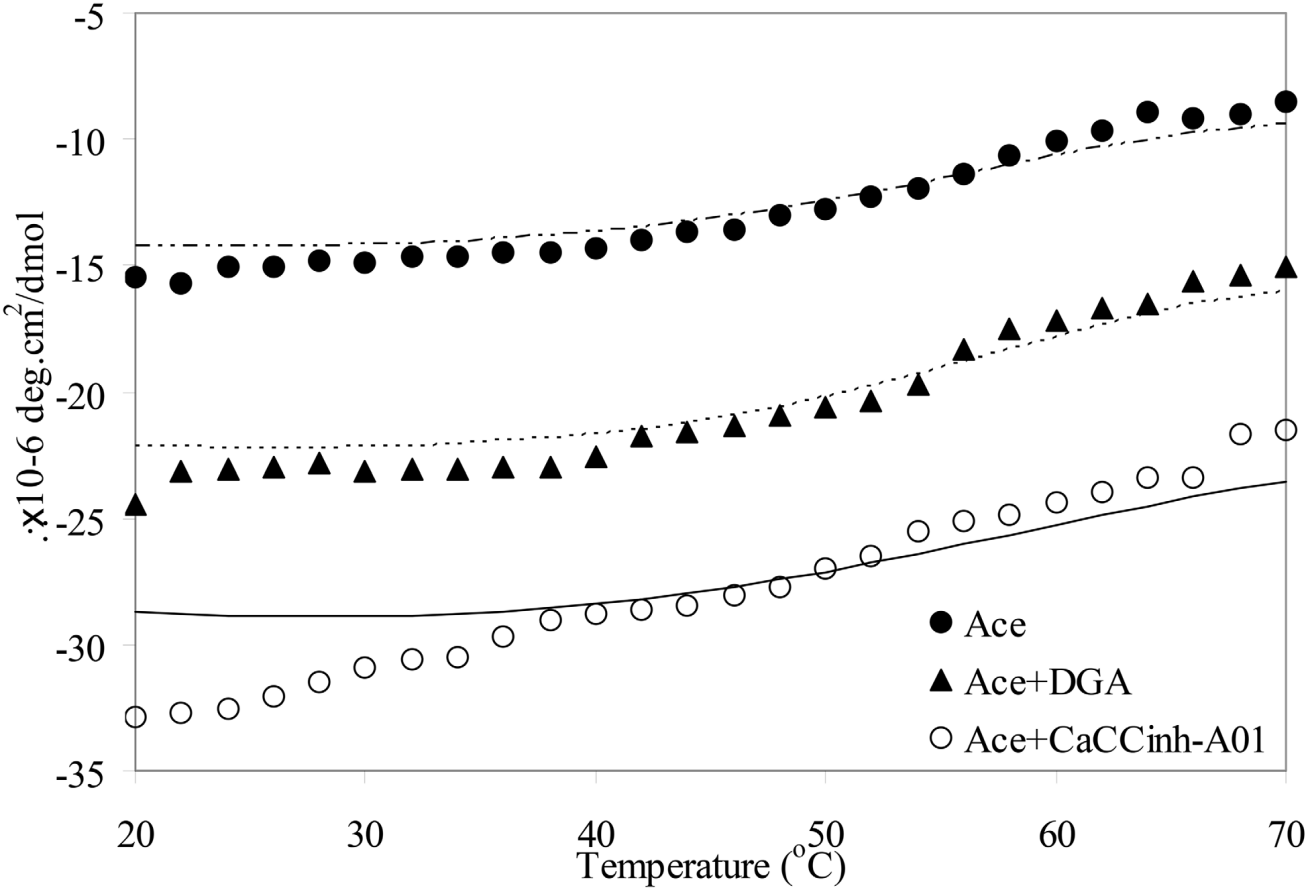
Temperature induced unfolding, measured using CD spectra, of Ace and Ace complexed with CaCC inhibitors.

Temperature induced unfolding of native Ace and Ace-small molecule complexes were measured from far-UV CD by plotting *θ*_222_ at different temperatures. The midpoint of the unfolding transition *T*_*M*_, as obtained from the sigmoidal fits of the plot of *θ*_222_ with temperature, was found to be 52°C for native Ace (Fig 3). The higher values of *T*_*M*_ for both Ace-DGA and Ace-CaCC_inh_-A01 complexes (55 and 57°C, respectively), compared to native Ace was indicative of the higher stability of Ace upon binding to CaCC_inh_-A01 and DGA. We excluded tannic acid for temperature induced unfolding as the protein lost its α-helical content upon binding to Ace.

### Small molecules induced fluorescence quenching of Ace

**Fig 4:**
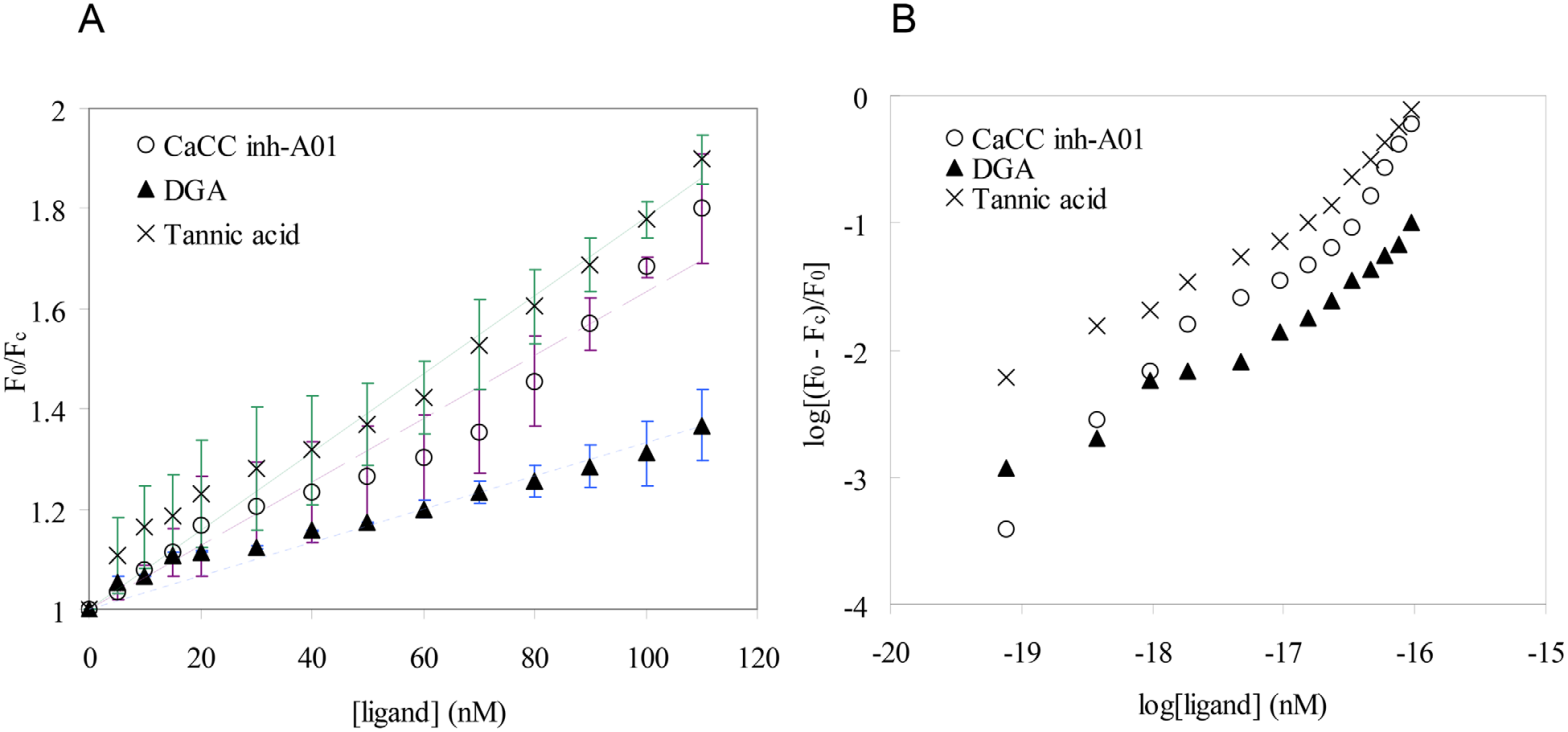
(A) Quenching of tryptophan fluorescence of Ace with CaCC inhibitors. (B) log [(Fo –Fc)/Fc] versus log [ligand] plot for the fluorescence quenching of Ace with CaCC inhibitors.

Quenching of tryptophan fluorescence is frequently used to study protein-ligand interaction [29,30]. Addition of CaCC inhibitors resulted in quenching of tryptophan fluorescence of Ace. The quenching data are presented as a Stern-Volmer plot, showing *F*_0_/*F*_c_ versus concentration of small molecules (Fig 4A). A plot of log[(*F*_0_−*F*_*c*_)/*F*_*c*_].versus log([*Q*]) for Ace-small molecules interaction using least-squares analysis gave the binding constants (Fig 4B).

The binding constants (*K*) as obtained from the intercepts on y-axis, along with binding stoichiometry (*n*) are given in Table 3. The binding constant obtained for Ace-tannic acid complex is less by almost one order of magnitude as compared to Ace-CaCC_inh_-A01 and Ace-DGA complexes. The standard free energy change (*ΔG* ^0^) for binding of CaCC inhibitors to Ace was determined from the relationship

$$\Delta G^{\text{0}}=-2\text{.}303RT\text{log}(K)$$ (8)

**Table 3:** Parameters of CaCC inhibitors binding to Ace obtained from tryptophan fluorescence quenching.

Parameters	Ace + CaCC_inh_-A01	Ace + DGA	Ace + tannic acid
**Binding constant (*K*, M** ^**-1**^ **)**	(1.4±0.9) × 10^7^	(1±0.03) × 10^7^	(3.7±1.02) × 10^6^
**Protein-small molecule stiochiometry (*n*)**	0.95 ± 0.3	0.85 ± 0.2	0.73 ± 0.27
**Binding free energy (Δ*G*, kcal.mol** ^**-1**^ **)**	-9.72 ± 1.4	-9.52 ± 1.2	-8.93 ± 1.0

The standard free energy of binding is slightly greater for CaCC_inh_-A01 and DGA than tannic acid complex.

### Effect of CaCC inhibitors short-circuit current (*I*_*SC*_) induced by Ace

**Fig 5:**
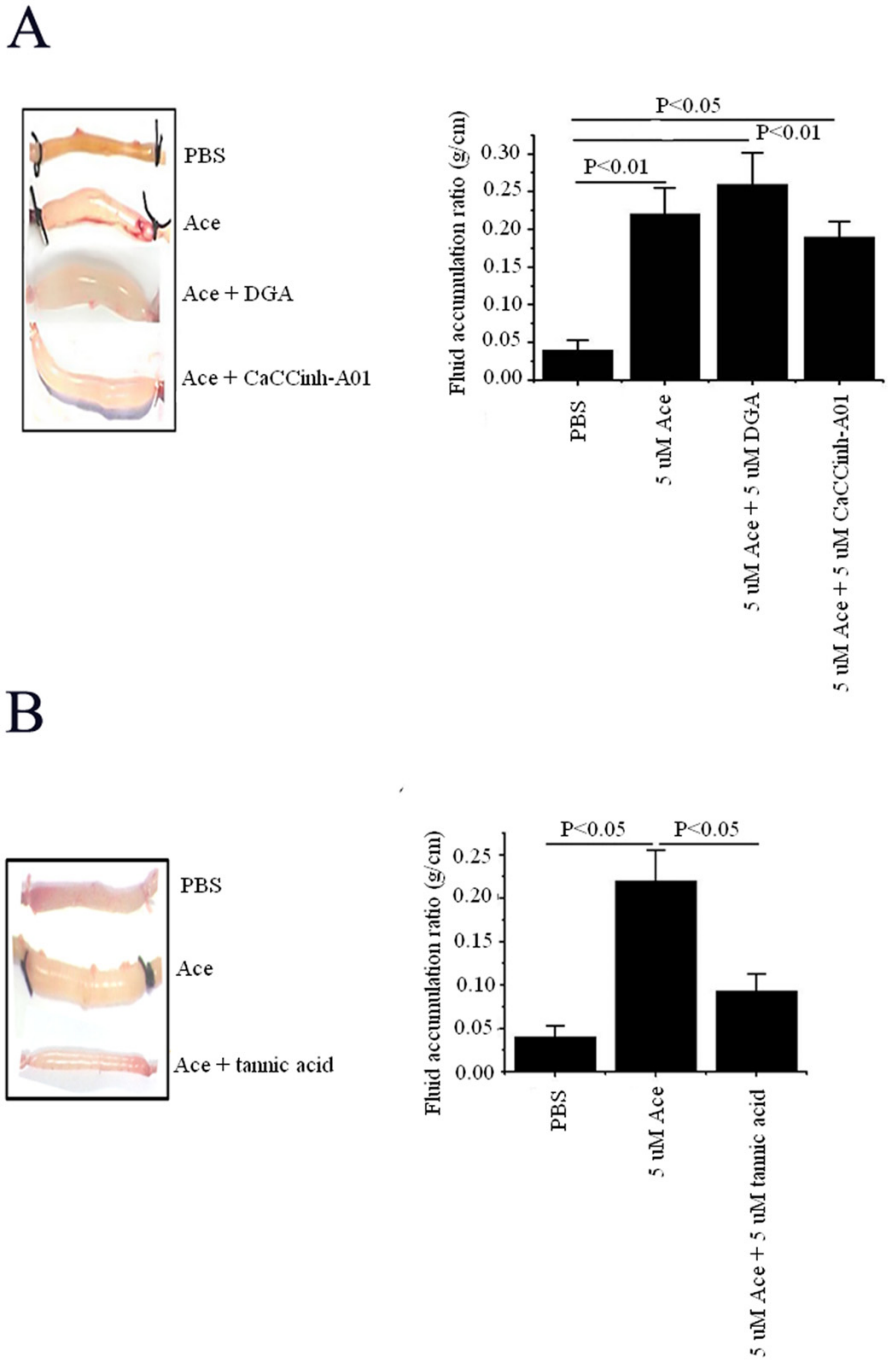
Effects of CaCC inhibitors on basal and Ace-stimulated short circuit current (*Isc*) in mouse tissue by Ussing chamber experiment, as described in the Materials and Methods. Basal (unstimulated) and Ace stimulated *Isc* was measured in the absence (control) and presence of (A) CaCC_inh_-A01 and (B) tannic acid. Two different concentrations of small molecules, low (5 μM) and high (100 μM), were used in each case and pre-incubated luminally before being treated with Ace toxin to the luminal side of the tissue. Results represent mean ± SEM of 6 tissue pairs; NS indicates the difference is not significant.

**Fig 6:**
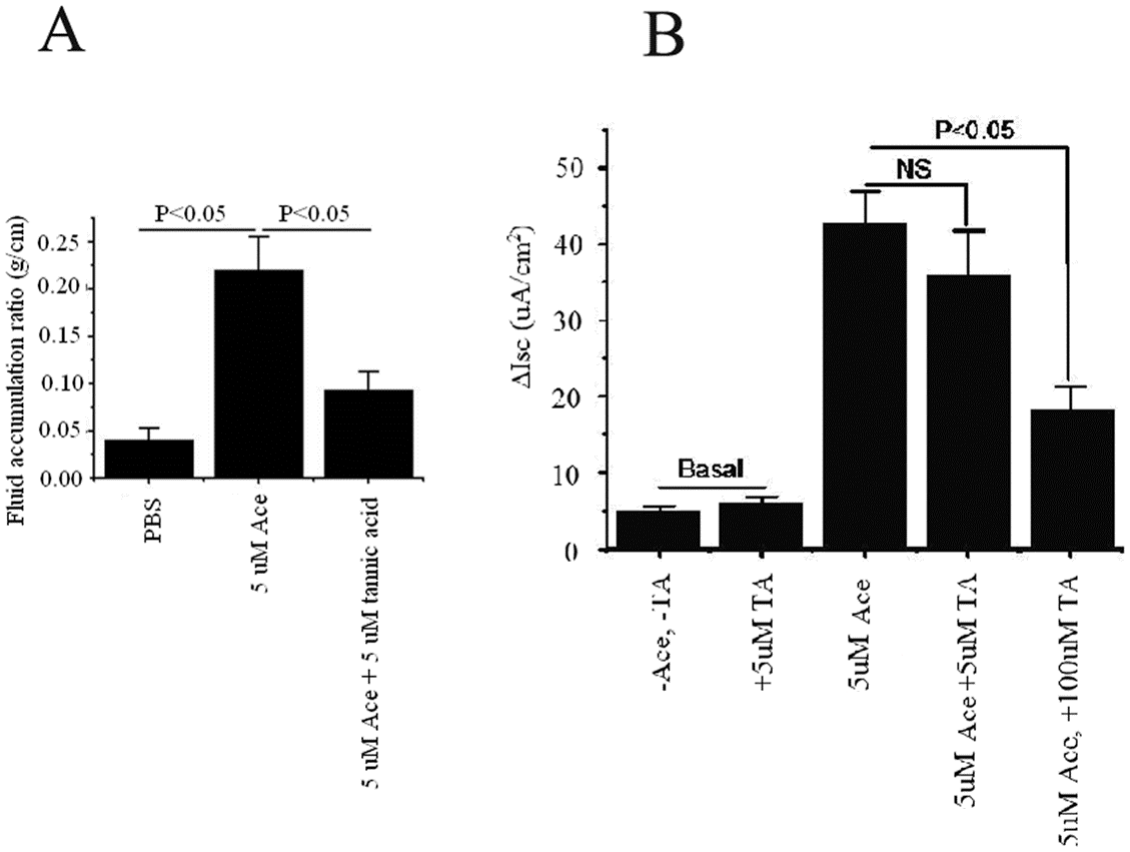
Effect of CaCC inhibitors on Ace stimulated intestinal fluid accumulation. Representative mouse ileal loops 6 h after luminal injection with Ace in the presence or absence of (A) DGA and CaCC_inh_-A01 and (B) tannic acid in 1:1 molar ratio (left panel). The right panel provides the bar graph showing the quantified averaged fluid accumulation in the loop experiment (n = 5–10). Values are mean ± SEM.

Ussing chamber experiment was performed in mouse tissue to find out the inhibitory effect of small molecules, CaCC_inh_-A01 and tannic acid on chloride channel before being stimulated by Ace. To prove that low concentration of small molecules did not have any effect on chloride channels but was effective enough to cause alteration of toxin structure (as found in ileal loop experiment, given below), tissues were pre-incubated by 5 μM as well as 100 μM of CaCC_inh_-A01. Low concentration of CaCC_inh_-A01 did not have any effect either on basal or Ace stimulated short-circuit current (*Isc*) (Fig 5A). However, a high concentration (100 μM) of CaCC_inh_-A01, which is believed to inhibit chloride channel, effectively inhibited Ace stimulated *Isc* [28]. Similar effect was also found with high concentration of tannic acid, while low concentration of the same was not able to inhibit basal as well as Ace stimulated *Isc* (Fig 5B). These results demonstrate that low concentration of CaCC_inh_-A01 and tannic acid has nothing to do with CaCC inhibition, rather they altered the structure of Ace, which in turn affected the degree of fluid accumulation in mouse ileal loop experiments (Fig 6).

### CaCC inhibitors attenuate Ace induced intestinal fluid accumulation

The efficacy of Ace in the presence of CaCC inhibitors was tested in an *in vivo* ligated mice ileal loop experiment. As shown in Fig 6A there was significant distension caused by fluid accumulation (FA) in Ace (5 μM) injected loop. As DGA and CaCC_inh_-A01 conferred structural stability to Ace, we reasoned that the administration of DGA and CaCC_inh_-A01 in combination with Ace, at 1:1 molar ratio, would cause more distension due to FA as compared to Ace injected loop. This indeed was the case that DGA exhibited additive FA of Ace, thereby enhancing FA ratio (Fig 6A). Simultaneous addition of CaCC_inh_-A01 and Ace did not exhibit additive effect on FA; however, it did not reduce the Ace stimulated FA either, possibly indicating a neutral effect on the toxin structure. On the contrary, administration of tannic acid in combination with Ace at 1:1 molar ratio effectively prevented FA in intestinal loops (Fig 6B). The result with tannic acid was along the expected line, as the molecule was found to destabilize Ace. Overall, our *in vivo* data are in agreement with the results obtained by biophysical experiments suggesting that DGA and CaCC_inh_-A01 stabilized, whereas tannic acid destabilized protein structure which affects the function of Ace.

### MD simulations

**Fig 7:**
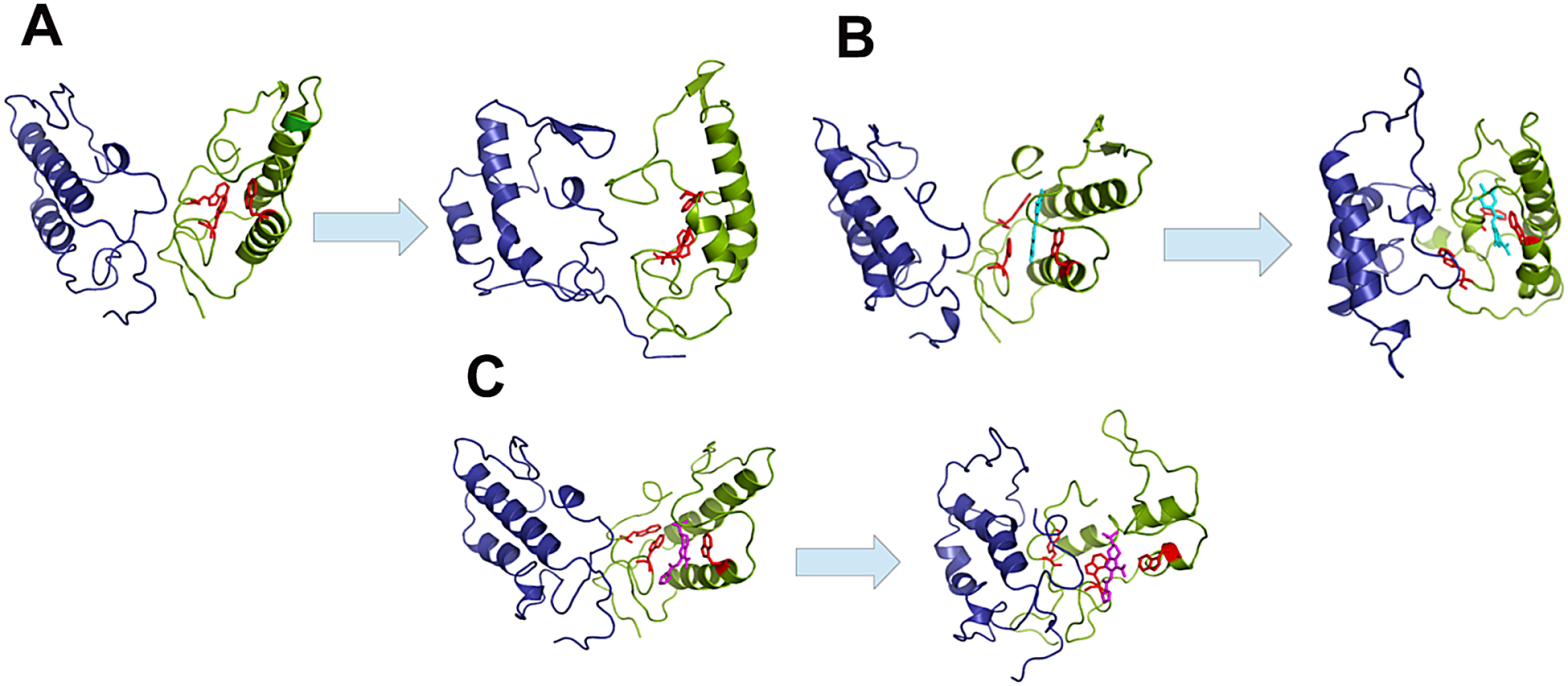
Structures of (A) Ace alone, and (B) complexed with DGA and (C) CaCC_inh_-A01; in each case the left panel indicates the start and the right panel the end of simulation time period. The A subunit of the Ace protein is shown in green cartoon and the B subunit is in blue: tryptophan residues 10, 17 and 38 are shown in red sticks. DGA is represented as cyan sticks (B), and CaCC_inh_-A01 as magenta sticks (C).

Ace in the uncomplexed state as well as when bound to DGA undergoes a significant amount of structural deviation as observed in Fig 7A. The protein has two chains A and B, and structural deviations occur in both subunits. At around ~7 ns, hydrogen bonds are formed between the residue Gln42 of subunit A and Gln151 of subunit B; additional hydrogen bonds across the interface are also formed between Lys43 (chain A) and Asp101 (chain B) (S3 Fig, S1 Movie). These interactions help to attain greater contacts at the dimeric interface of Ace. The interface area of the starting structure is 903 Å^2^, which increases to 1912 Å^2^ at the end of 20 ns.

For both the complexes formed with DGA and CaCC_inh_-A01 inhibitor, deviation is spread all over the structure as well. In the DGA-protein complex, the two subunits come closer after simulation (Fig 7B, S2 Movie). Trp38 is in contact with the ligand throughout the simulation; the hydrogen bonds between the residues Asp46, Met47, Asp75 (from subunit A) and the ligand are also observed. Additional hydrogen bonds are also formed with Asp46 (chain A), and Tyr104 (chain B) of the protein. The hydrophobicity of the binding site (the residues within 4 Å of the ligand were considered) increases towards the end of simulation, with new interactions being formed with Leu2, Leu11, Phe48 and Ty104. On the other hand, CaCC_inh_-A01 in the complex undergoes quite a few rotational changes, affecting the shape of the molecule, leading to a RMSD larger than what was seen for DGA (Table 4).

**Table 4:** Binding energetics of DGA and CaCCinh-A01 with Ace from MD simulation^†^.

Protein/complex	Average ΔG_binding_ (kcal.mol^-1^)	Interface area (Å^2^)	RMSD (Å) of ligand	RMSD (Å) of protein
**Ace**	-	1912	-	8.3
**Ace-DGA**	-33± 3	2020	0.3	8.0
**Ace-CaCC**_**inh**_ **-A01**	-37 ± 3	2151	1.1	6.8

^**†**^ Interface area and RMSD are calculated after 20 ns of simulation

**Fig 8:**
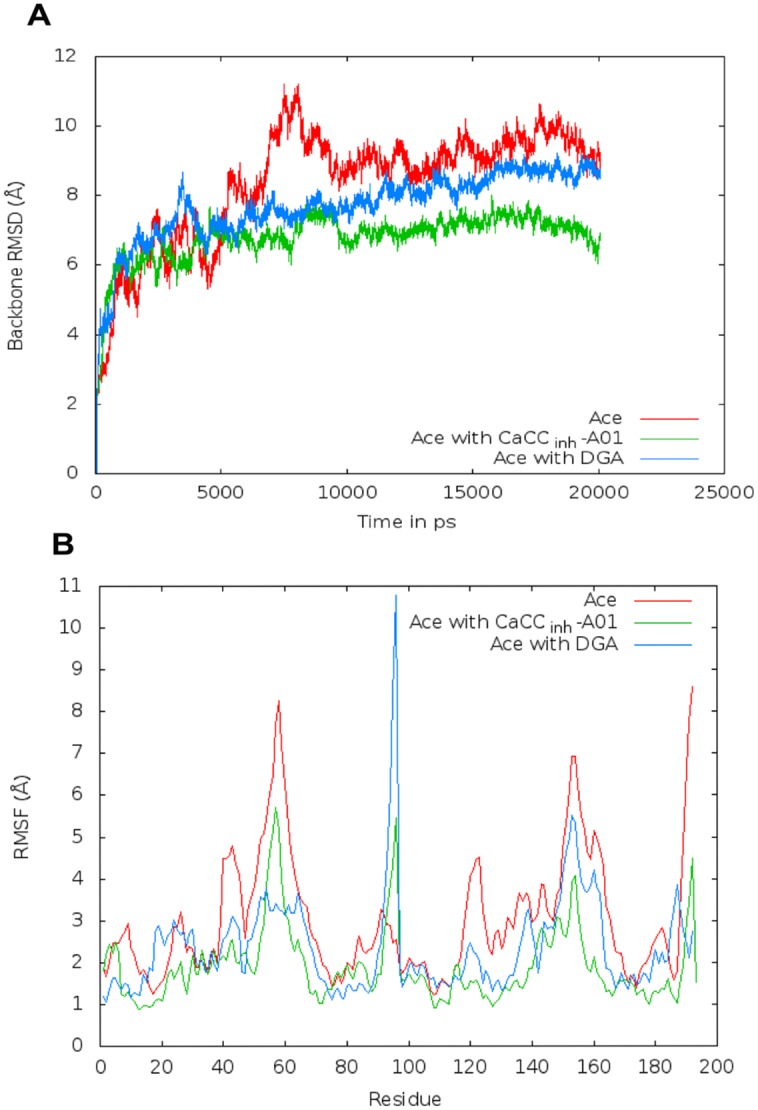
The backbone (A) root mean square deviations (RMSD) and (B) root mean square fluctuation (RMSF) of uncomplexed Ace along with the protein bound to DGA and CaCC_inh_-A01. Both the subunits are considered in (A); in (B) residue numbers 1–96 correspond to chain A, and 97–192 to chain B.

Three Trp residues marked in red sticks (Fig 7C, S3 Movie) that are associated with the binding of ligands (experimentally Trp quenching is observed after complexation with both DGA and CaCC_inh_-A01) come closer to the ligands after 20 ns of simulation. CaCC_inh_-A01 inhibitor mainly interacts with the residues of chain A, forming hydrogen bonds with Leu18, Gln35 and Trp38. Only Tyr104 of the other chain is seen to form hydrogen bonds with the ligand around 5 ns, and remains within interacting distance from the ligand till the end of simulation. The binding site becomes increasingly hydrophobic, and additional interactions with Trp17, Leu18, Val19, Phe37, and Leu74 are observed. The dimeric interface area increases from 959 to 2151 Å^2^ for Ace-CaCC_inh_-A01 complex, 1124 to 2020 Å^2^ for DGA bound structure at the end of simulation. The compactness of a structure can be estimated from the radius of gyration, Rg. Both the complexes attain a more compact structure as seen from the Rg value, which decreases from 20 Å in the uncomplexed structure to 18 Å in the complex. Moreover, the average Δ*G*_binding_ values (Table 4) indicate that CaCC_inh_-A01 has a higher affinity (-37 kcal/mol) towards the protein than DGA (-33 kcal/mol). The backbone root mean square deviations (RMSD) profile using the main-chain atoms (C, C^α^ and N), versus time period of simulation, and the root mean square fluctuation (RMSF) (averaged over time) for each constituent residue are presented in Fig 8. It can be seen that both the values are in general lower for the complexes as compared to the free Ace.

As the RMSD for the protein alone was rather high (Table 4), we wanted to check if the secondary structures were robust during simulation. Indeed this was the case—the average percentage helicity did not change much (33±3% for Ace, and 36±3% and 30±3% for the complexes with DGA and CaCC_inh_-A01, respectively) (S4 Fig). The RMSD of the four major helices are in the range 1.1±0.3 to 2.9±0.6 Å. It may be mentioned that large RMSD value (for the overall structure) is not unusual in simulations carried out with homology models [31], the retention of secondary structural features here would indicate the legitimacy of the model.

## Discussion

Activation of chloride channel(s) and inhibition of electroneutral sodium-chloride absorption is a pivotal step in the pathogenesis of secretory diarrhea, caused by *V*. *cholerae* infection [32]. Evidence from T84 cells monolayer as well as from animal models indicates that Ace protein of *V*. *cholerae* stimulates Ca^2+^ dependent secretion of Cl^-^/HCO_3_^-^ from enterocytes into the intestinal lumen, creating a driving force for sodium and water secretion [33]. Ace or Ace analogs is/are predicted to be of clinical benefit to treat diseases involving insufficient anion transport, such as CF [16]. Therefore, inhibitors and activators of chloride channels have potential application as anti-secretory therapy in cholera as well as in restoring apical membrane chloride permeability in epithelia, lacking normal chloride channel function. Literature reports various small molecules modulating the function of chloride channels as an attractive candidate for disease therapy [8], but how these small molecules affect the structure and hence the function of a particular toxin remains unexplored. In this context, we investigated the role of such small molecules on the structure/function of Ace, which could be of therapeutic benefit in gastrointestinal disorder like diarrhea without targeting host secretory mechanism, such as intestinal chloride channel.

Biophysical studies revealed disruption of the secondary structure of Ace after treatment with tannic acid. This was further confirmed from *in vivo* experiment, showing administration of Ace along with tannic acid in 1:1 molar ratio prevented accumulation of fluid in ligated mice ileal loop, in contrast to Ace alone (Fig 6B). Electrophysiology assay in Ussing chamber using mouse tissue pre-incubated with 5 μM tannic acid before being stimulated by Ace, showed no inhibition and had no effect on basal as well as Ace stimulated *Isc*, indicating that the reduction of fluid accumulation in ileal loop was not due to the inhibition of calcium activated chloride channel, but due to the degradation of toxin structure. CD experiments indicated loss of α-helical content of Ace upon treatment with tannic acid. However, high concentration of tannic acid, which is considered to be specific for CaCC inhibition, significantly reduced Ace stimulated *Isc* suggesting its inhibitory effect on CaCC. The other two small molecules, DGA and CaCC_inh_-A01, in contrast, stabilize the protein structure, as evident from far-UV CD (Table 2). As expected, additive distension of mice ileal loops was observed upon injecting Ace along with DGA or CaCC_inh_-A01 (Fig 6). Interestingly CaCC_inh_-A01 had similar effect on *Isc* as that of tannic acid. We performed this experiment only with CaCC_inh_-A01 because CaCC_inh_-A01 has greater binding affinity for the receptor compared to DGA, in a sense that the greater hydrophobic bulk in the ligand might facilitate better binding in case of CaCC_inh_-A01 as presented in Table 4. Quenching of tryptophan fluorescence of Ace was observed upon binding to these CaCC inhibitors and binding constants obtained were indicative of quite strong binding of the ligands to the protein (Table 3). This observation was further corroborated from molecular docking and simulation studies, which indicate close proximity of tryptophans to both the ligands (Fig 7).

Results of MD simulations of the free protein and the complexes with two inhibitors indicate that Ace by itself is very flexible (Fig 7). Ligand binding reduces the flexibility of Ace especially in case of DGA binding, as evident from the RMSD values (Fig 8A, Table 4) as well as from the RMSF profile (Fig 8B); an increase in the interface area indicates that the dimeric structure is stronger in presence of the ligands (Table 4). A slight increase in helicity is also observed for DGA bound complex, although the average helicity changes very little for all the three structures (S4 Fig). In a recent work, the effect of CaCC_inh_-A01 and DGA on the dimerization of ANO1 was studied [15]. The ligands did not affect the dimerization of ANO1; instead a different method of degradation of the protein was implicated, which had their origins in hydrophobic interactions of the ligands with the receptor, at the expense of native protein contacts. Our simulations also indicate that the binding sites for both the ligands take on an increasing hydrophobic environment and the protein becomes more compact as evident from the R_g_ values. All three compounds used in the present study contains carboxylic acid group, but the hydrophobic bulk of CaCC_inh_-A01 or tannic acid is absent in DGA [15]. Tannic acid, which causes the maximum disruption of toxin structure, has significantly high hydrophobic surface area (due to the presence of large number of aromatic rings). These may form π-stack with aromatic amino acid residues (especially Trp and Tyr) of Ace, thereby compromising the structure [34,35].

In the present work, we have identified tannic acid as an inhibitor which destabilizes the structure and hence the function of Ace. On the contrary, the role of DGA in stabilizing the structure of Ace may be of therapeutic benefit in epithelia lacking normal CFTR function that cause lung disease like CF. In summary the biophysical data, and results from *ex vivo* and *in vivo* experiments suggest opportunities for bacterial toxin targeted therapeutic for diarrhea and other gastrointestinal disorders. The uniqueness of the present study lies in the fact that the modulation of the structure of a toxic protein with low concentration of CaCC inhibitor, without affecting the host chloride channel, may hold considerable promise for treating diarrhea and epithelial disorders lacking chloride channel function.

## Supporting Information

S1 Fig Chemical structures of small molecules. (DOC)

S2 Fig The model of Ace and the sequence alignment used for homology modelling. (DOC)

S3 Fig Cartoon representation of hydrogen bonds. (DOC)

S4 Fig The percentage helicity of the uncomplexed Ace along with the complexes against time. (DOC)

S1 Movie Dynamics of uncomplexed Ace. (MPG)

S2 Movie Dynamics of Ace complexed with CaCC_inh_-A01. (MPG)

S3 Movie Dynamics of Ace complexed with DGA. (MPG)

## Abbreviations

Ace: Accessory cholera enterotoxin
CaCC: calcium-activated chloride channel
CF: cystic fibrosis
CFTR: cystic fibrosis transmembrane conductance regulator
CTX: cholera toxin
DGA: digallic acid
DMSO: dimethyl sulfoxide
IPTG: isopropyl-β-D-thiogalactopyranoside
MD: molecular dynamics
Ni-NTA: Nickel nitriloacetic acid
PDB: Protein Data Bank
PMSF: phenyl methyl sulfonyl fluoride
Rg: radius of gyration
RMSD: root mean square deviations
RMSF: root mean square fluctuation
