# Supplementary figures and images for: Effects of Small Molecule Calcium-Activated Chloride Channel Inhibitors on Structure and Function of Accessory Cholera Enterotoxin (Ace) of Vibrio cholerae
Source: PLoS One. 2015 Nov 5;10(11):e0141283. doi: 10.1371/journal.pone.0141283 (PMC4634967; doi:10.1371/journal.pone.0141283)

**S1 Fig.** Chemical structures of (a) tannic acid, (b) CaCCinh-A01 and (c) digallic acid.


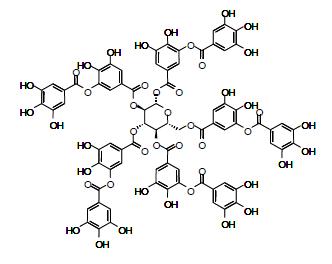


(a)


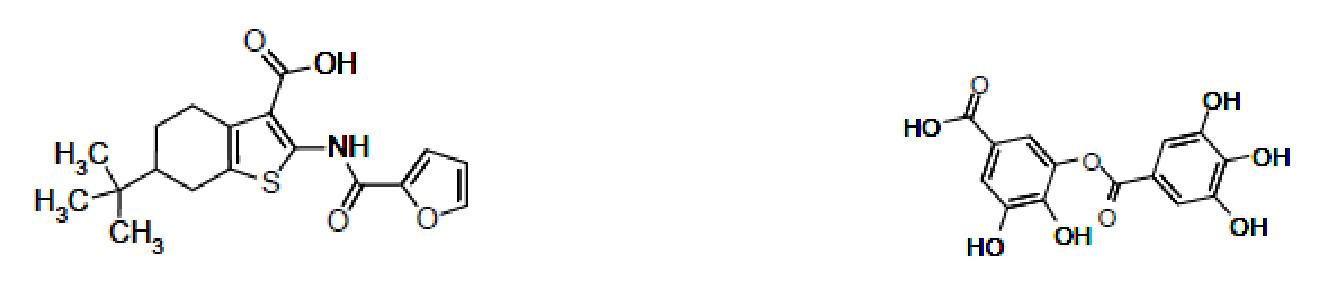


(b) (c)

Supplement: S1 Fig — (DOC) [file pone.0141283.s001.doc]
