# Supplementary material for: Effects of Small Molecule Calcium-Activated Chloride Channel Inhibitors on Structure and Function of Accessory Cholera Enterotoxin (Ace) of Vibrio cholerae
Source: PLoS One. 2015 Nov 5;10(11):e0141283. doi: 10.1371/journal.pone.0141283 (PMC4634967; doi:10.1371/journal.pone.0141283)
Supplement: S2 Fig — (DOC) [file pone.0141283.s002.doc]

**S2 Fig.** The model of Ace and the sequence alignment used for homology modelling. **(a)** The dimeric structure of Ace (residues 1-96 in subunit A, 97-192 in B); helices (red) are named as H1 (Trp26 to Met40), H2 (Ser66 to 85), H1’ (Trp121 to Met136) and H2’ (Ser162 to Leu181). (b) Alignment of the Ace sequence with the template zhaoermiatoxin from *Zhaoermia mangshanensis* snake venom. Conserved residues are denoted by asterix (*). (c) Data indicating the validation of the structure. The data in (b) and (c) are taken from *Biochemistry* 2011; 50: 2962-2972*.* [Ref # 7].

(a)


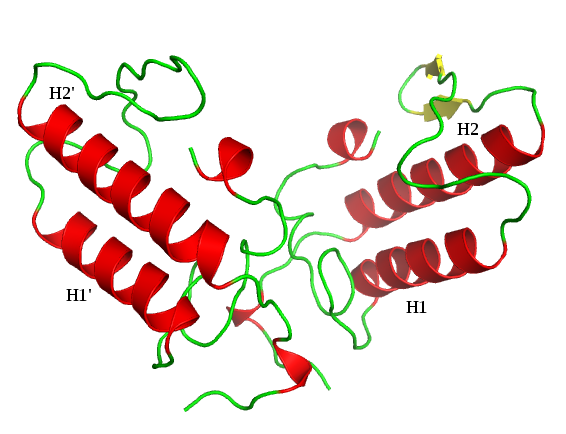


(b) CLUSTAL 2.1 multiple sequence alignment

| 2PH4_A|PDBID|CHAIN|SEQUENCE | SLIELTKMVFQETGKNPVTYYTLYGCNCGVGRRGKPKDATDRCCFVHRCC | | | | | | 50 |
| --- | --- | --- | --- | --- | --- | --- | --- |
| tr|C3M620|C3M620_VIBC3 | -------MLMMD----PLYDWLIDGFTWLVIKLG--IMWIESKIFVIQFF | | | | | | 37 |
|  |  | *:: : | *: | : : * . | * : * | : ** : |  |
| 2PH4_A|PDBID|CHAIN|SEQUENCE | YKKLTGCDPKKDRYSYSWENKAIVCGEKNPCLKELCECDKAVAICLRKNL | | | | | | 100 |
| tr|C3M620|C3M620_VIBC3 | WEMSQKVIDMFTIYPLIQQAIDMLS-PQYSGFLFFLGLDQALAIVLQALM | | | | | | 86 |
|  | :: | *. | : | ::. | : . : : | *:*:** *: : |  |
| 2PH4_A|PDBID|CHAIN|SEQUENCE | GTYDKNYRFTMKFLCDKPEKC 121 | | | |  |  |  |
| tr|C3M620|C3M620_VIBC3 | T------ | RFALRALNL | ----- | 96 |  |  |  |
|  |  | **::: * |  |  |  |  |  |

(c) Stereochemical evaluation of backbone φ and ψ dihedral angles by PROCHECK revealed that 83.9, 10.9, 4.1, and 1.1% of residues were within the most favored region, additionally allowed region, generously allowed region, and disallowed region, respectively, of the Ramachandran plot. Similarly, WHATIF revealed that the root-mean-square Z score for bond lengths, root-mean-square Z score for bond angles, ω angle restraints, side chain planarity, improper dihedral distribution, and outside distribution were 0.912, 1.368, 0.760, 0.407, 0.999, and 1.351, respectively for the modeled structure, which are all positive values (positive is better than average).
