# Supplementary material for: Effects of Small Molecule Calcium-Activated Chloride Channel Inhibitors on Structure and Function of Accessory Cholera Enterotoxin (Ace) of Vibrio cholerae
Source: PLoS One. 2015 Nov 5;10(11):e0141283. doi: 10.1371/journal.pone.0141283 (PMC4634967; doi:10.1371/journal.pone.0141283)

**S3 Fig.** Cartoon representation of hydrogen bonds (shown as dashed lines) formed between residues Gln42, Lys43 (in A subunit) and Gln151, Asp101 (B subunit) after 7 ns and which remain till the end of simulation. Two subunits of the protein are in green and blue and the residues are shown in sticks colored by the elements. Hydrogen bonds are formed between O of Gln42 and NE2 of Gln151, NZ of Lys43 and O of Asp101.


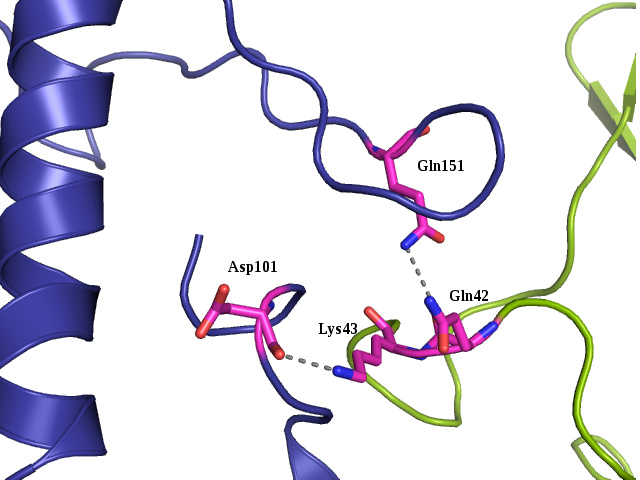

Supplement: S3 Fig — (DOC) [file pone.0141283.s003.doc]
