# Supplementary material for: Effects of Small Molecule Calcium-Activated Chloride Channel Inhibitors on Structure and Function of Accessory Cholera Enterotoxin (Ace) of Vibrio cholerae
Source: PLoS One. 2015 Nov 5;10(11):e0141283. doi: 10.1371/journal.pone.0141283 (PMC4634967; doi:10.1371/journal.pone.0141283)

**S4 Fig**. The percentage helicity of the uncomplexed Ace along with the complexes formed with the two ligands are plotted against time.


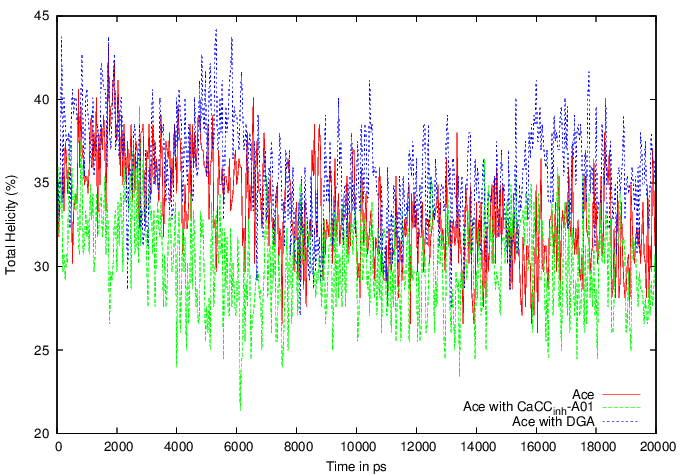

Supplement: S4 Fig — (DOC) [file pone.0141283.s004.doc]
